# 3D imaging and printing in pelvic colorectal cancer: ‘The New Kid on the Block’

**DOI:** 10.1007/s10151-018-1922-y

**Published:** 2019-01-17

**Authors:** C. Kontovounisios, P. Tekkis, F. Bello

**Affiliations:** 1 Department of Colorectal Surgery, Chelsea and Westminster Hospital, London, UK; 2 Department of Colorectal Surgery, Royal Marsden Hospital, London, UK; 3 Department of Surgery and Cancer, Imperial College, London, UK; 4 Simulation and Modelling in Medicine and Surgery, Centre for Engagement and Simulation Science, Imperial College London, London, UK

Imaging modalities aid clinicians in diagnosis, function assessment, gauging response to treatment and assisting pre-/intra-/postoperatively in surgery.

The primary goal of pelvic cancer surgery is to perform radical resection to achieve complete tumour clearance (R0), leading to a significant increase in survival. Proper patient evaluation and surgical planning are necessary to achieve these goals. A wide range of pre-operative planning techniques can be applied, some of which allow surgeons to practice and refine the procedure, improving efficiency in the operating room, shortening operative time, and reducing incidence of iatrogenic complications.

**Fig. 1:**
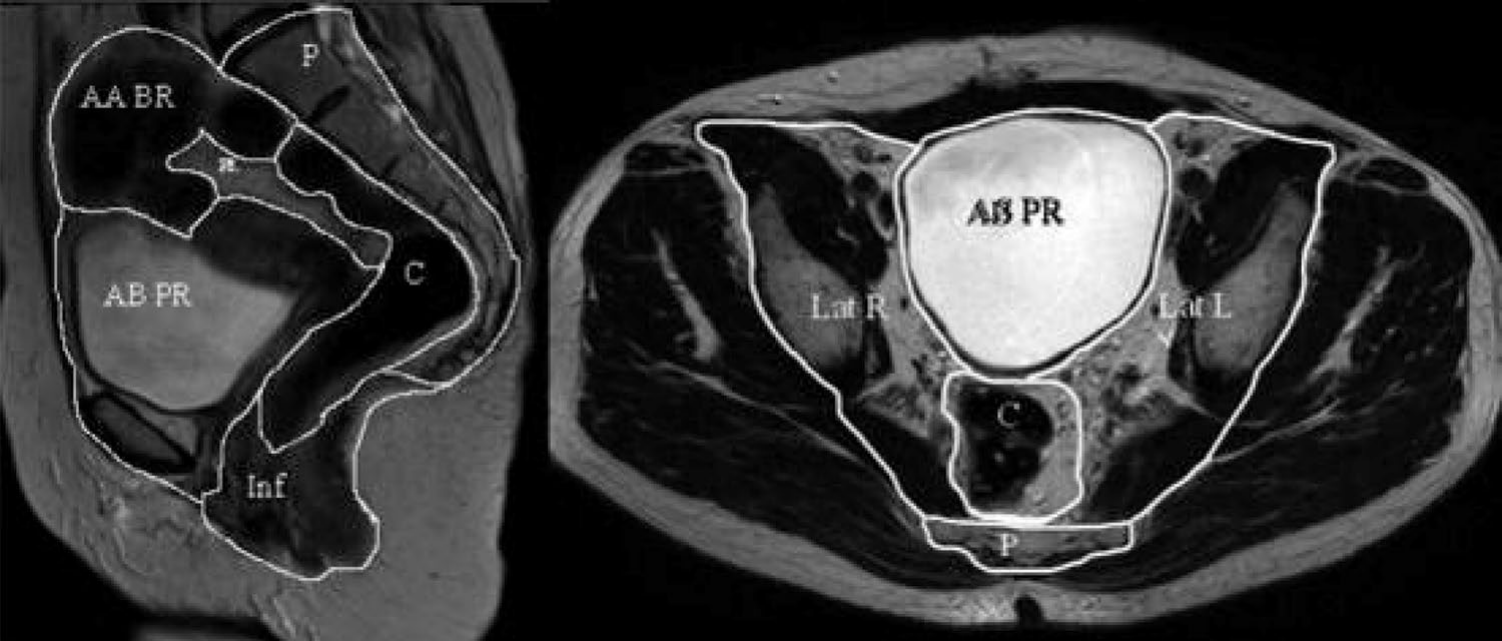
Sagittal and axial T2-weighted magnetic resonance imaging (MRI) images showing the seven intrapelvic compartments introduced in [1]

Magnetic resonance imaging (MRI) is an important diagnostic tool in identifying relevant anatomy and staging in rectal cancer. Findings on MRI confer major implications for diagnosis and management, including surgical planning, prediction of adverse surgical outcomes, selecting patients for neoadjuvant therapy and eligibility for surgery. MRI is also accurate at identifying invasion of cancer into adjacent pelvic compartments and structures [1]. Existing pro forma (standardized imaging criteria) can be used to interpret MRI for colorectal cancer (CRC), but these require specialist training (Fig. 1).

The functional anatomy of the pelvic organs and muscles work synergistically to maintain continence. Dysfunction and disruption of these intricate inter-related structures through disease or surgical intervention result in debilitating conditions such as faecal/urinary incontinence, obstructive defecation syndrome and prolapse.

Three-dimensional (3D) imaging can be used to construct ‘virtual’ 3D objects that are visualised and manipulated on a computer screen or Head-Mounted Display (HMD), whilst 3D printing can be used to create 3D objects under computer control using successive layers of material. First described in the 1980s, 3D printing technology has established commercial and research applications using materials such as metal, plastic, or ceramic [2, 3]. However, there has been a delay of 3D modelling use in general surgery, and especially in pelvic surgery, compared to other specialties. Soler et al. [4] published the first fully automatic 3D reconstruction liver model, allowing surgeons to build an anatomic segmentation of the liver based on the Couinaud subsegments, with delineation of the hepatic and portal veins. These tools provide virtual planning of liver resection by considering the spatial relationship between the tumour and the hepatic vascular trees, as well as the size of the future liver remnant. Another important step was the application of frameless stereotactic liver surgery in tumour resection. Similar to neurosurgery, an interactive image-guided surgery system for liver surgery was evaluated for accurate instrument tracking.

Recently, Sahnan et al. [5] published their experience of 3D modelling in pre-operative planning of perianal fistula. Surgeons can review images prior to and during the procedure with benefits in operating time and outcomes. They choose the type of operation best suited to the patient by demonstrating the intersphincteric complexity that may preclude a ligation of the fistula tract procedure, or the secondary extensions that would doom a fistula plug to failure. The same team has used 3D modelling in surgical planning for transanal total mesorectal excision (TaTME), demonstrating how this technique is feasible and can be derived from manipulation of standard two-dimensional (2D) MRI DICOM images [6].

Despite these rapid advances in the operative application of 3D technology, surgical simulation and planning based on 3D models are not yet routinely used. There are several reasons for its slow adoption. A crucial consideration is the segmentation process (partitioning or labelling of a digital image into multiple segments of interest), which is typically time consuming and highly operator dependent, yet is the initial step in 3D model generation.

**Fig. 2:**
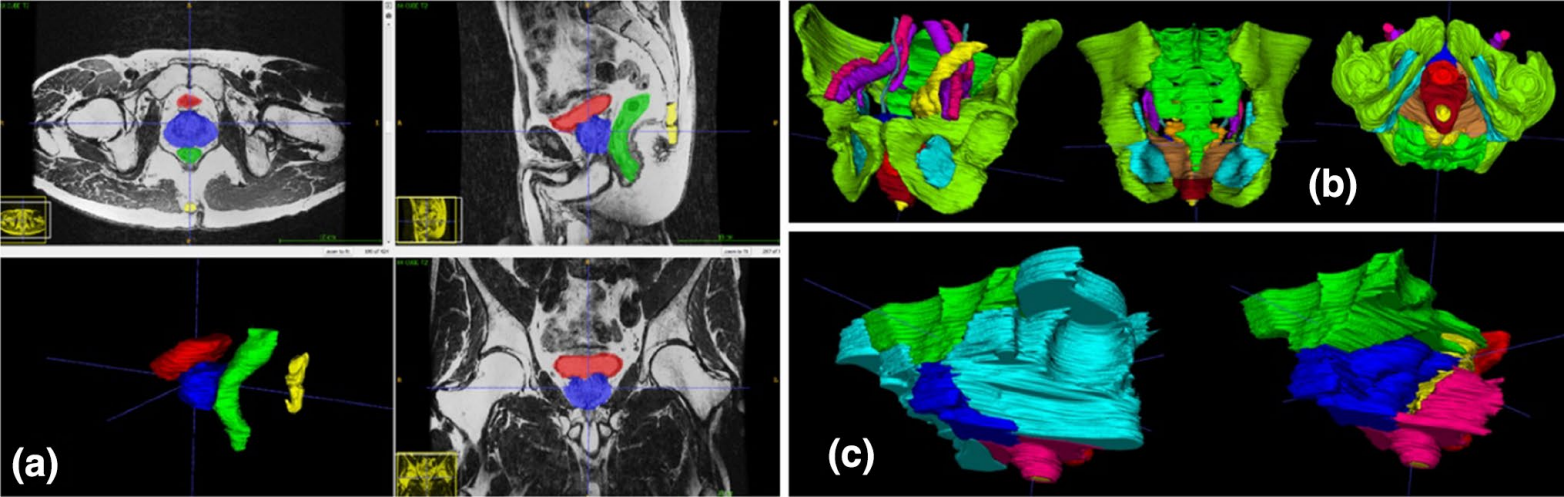
Segmented 3D anatomy and compartments of healthy male volunteer. **a** Organ delineation in MRI. **b** 3D anatomy. **c** 3D compartments with (left) and without (right) lateral compartments

Working closely with radiologists, we have established rigorous and reproducible segmentation criteria of male and female pelvic anatomy, and pelvic compartments from MRI scans that streamline the process and ensure the reliability of the resulting 3D models, paving the way for building a substantial database and for the development of fully automated, data-driven segmentation tools. Particular emphasis was given to colorectal cancer (CRC), anatomy, and the boundaries and contents of the central compartment (rectum, intra/extra-luminal, perirectal fat, mesorectum), due to its medical and surgical importance, specifically in total mesorectal excision (TME) (Fig. 2).

**Fig. 3:**
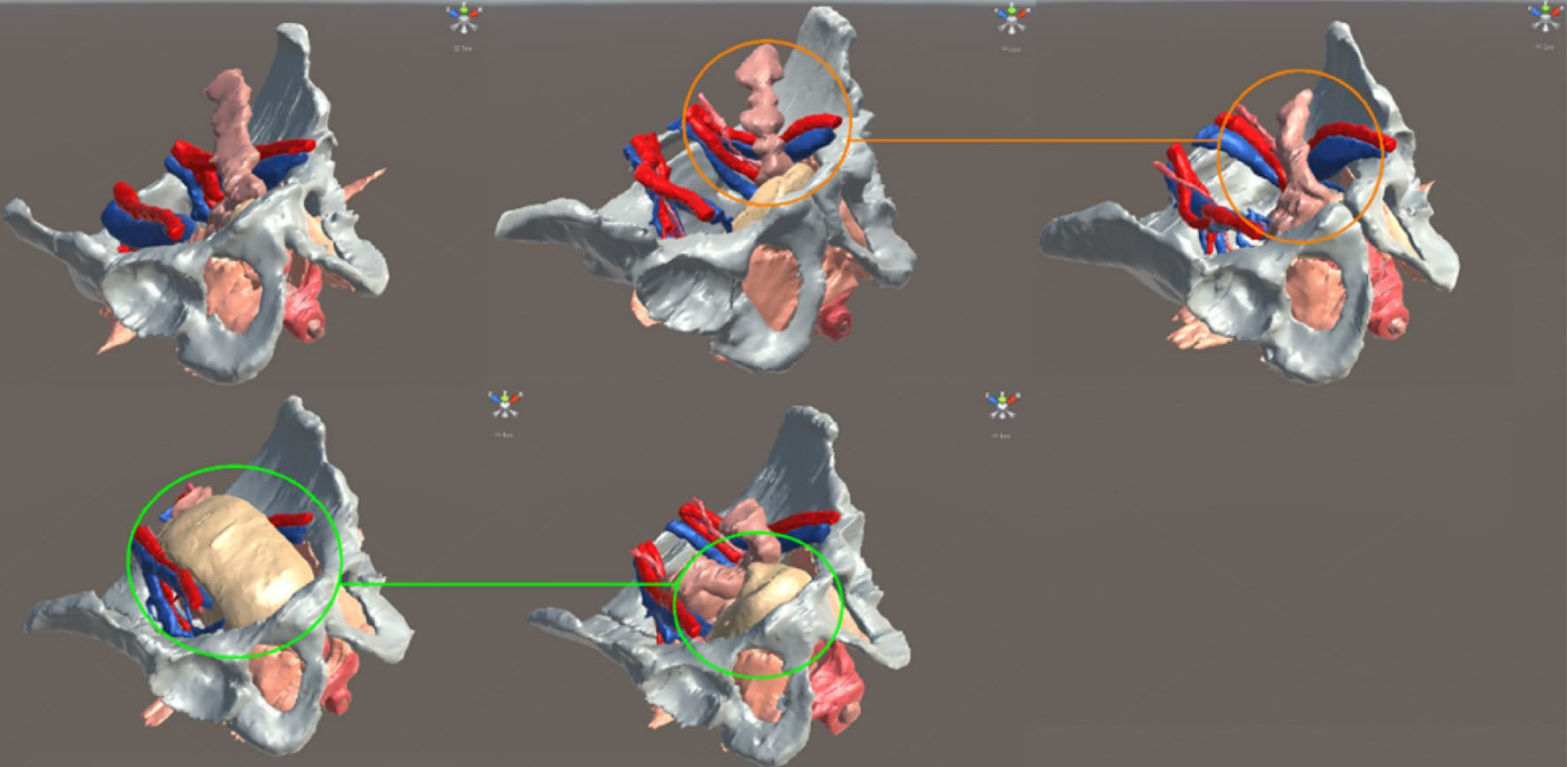
3D models of five healthy male volunteers illustrating anatomical variation (orange) and organ distension (green)

A total of ten pelvic MRIs from healthy volunteers (five male/five female) have been successfully segmented with our segmentation criteria (Fig. 3), using well-documented software tools (ITK-SNAP, Meshlab) to segment and generate the three-dimensional models. We plan to extend this number and build a database of 3D models of cancer patients and patients with pelvic floor disorders, exploring its use for surgical planning, pre-operative staging, simulation of dynamic function of pelvic floor structures, enhancing patient interaction, understanding and participation in decision-making, as well as medical and surgical education.

We believe that the use of 3D models can provide doctors with more and better informed choices for surgical planning and consulting patients. Carefully constructed from imaging data, they offer a true-size, detailed representation for surgeons to use, making pre-operative planning more direct and accurate. 3D models can be used as an additional tool in the surgical planning for organ/compartment preservation, where fibrosis or disease post-chemoradiotherapy cannot be adequately estimated with current MRI models. They may also improve surgical efficacy and safety through intraoperative augmented reality and can support the simulation of pelvic organs and their function, with the potential for clinical, educational and engagement (clinicians and patients) applications in the future.

The work is very timely from both a CRC and technology perspective. CRC prognosis and survival are based on accurate initial staging. The use of 3D models as an additional tool in staging will improve accuracy and surgical planning. The innovation leverages recent advances in 3D graphics and visualisation, 3D printing and virtual/augmented reality. In the short term, we expect the work to result in adopted guidelines for the building and routine use of 3D models of pelvic anatomy. This will be followed by medium-term technological developments enabling automated 3D model generation, large-scale 3D pelvic anatomy databases, application of machine learning approaches, clinical translation into CRC surgical planning and dynamic function simulation.
